# Isolation, Identification, and Drug Sensitivity Test of *Pseudomonas aeruginosa* from Cynomolgus Monkey (*Macaca fascicularis*)

**DOI:** 10.3390/vetsci12070636

**Published:** 2025-07-03

**Authors:** Heling Li, Ziyao Qian, Yulin Yan, Hong Wang

**Affiliations:** 1 State Key Laboratory of Primate Biomedical Research, Institute of Primate Translational Medicine, Kunming University of Science and Technology, Kunming 650500, China; lihl@lpbr.cn (H.L.); qziyao_7yr@163.com (Z.Q.); 2 Yunnan Key Laboratory of Primate Biomedical Research, Kunming 650500, China; 3 College of Veterinary Medicine, Yunnan Agricultural University, Kunming 650201, China

**Keywords:** cynomolgus monkey, *Pseudomonas aeruginosa*, identification, drug sensitivity test

## Abstract

In this study, we isolated and identified bacteria from the feces of a diarrheal cynomolgus monkey. The results showed that the isolated strain was *P. aeruginosa*, named PA/CM-101101. Morphological observations indicated that when cultured on Luria–Bertani (LB) nutrient agar at 37 °C for 24 h, the strain formed smooth, slightly elevated colonies with neat and wavy edges. On acetamide agar at the same temperature and duration, the colonies appeared flat with irregular edges and a faint pink periphery, while the medium changed to rose-red; in LB broth at 37 °C for 24 h, the medium became turbid and yellowish-green. Gram staining revealed that it was negative and rod-shaped, without sporulation characteristics. The 16S rRNA gene sequence analysis showed that the sequence identity of the strain shared more than 98.4% similarity with 11 strains of *P. aeruginosa* from various sources in GenBank. The animal toxicity test showed that it had a strong pathogenic effect on mice. The results of drug sensitivity tests showed that strain PA/CM-101101 was sensitive to amikacin, azithromycin, cefoperazone, ceftazidime, ceftriaxone, ciprofloxacin, gentamicin, imipenem, levofloxacin, meropenem, norfloxacin, ofloxacin, and polymyxin B; however, it displayed resistance to ampicillin, cefadroxil, cefazolin, erythromycin, and vancomycin. The research findings provide valuable insights for diagnosis and treatment strategies for cynomolgus monkeys. It also provides a reference for molecular epidemiological studies. To our knowledge, this is the first time *P. aeruginosa* isolated from the diarrhea feces of cynomolgus monkey has been reported.

## Simple Summary

*Pseudomonas aeruginosa* (*P. aeruginosa*) is an aerobic, non-fermentative, oxidase-positive, small Gram-negative bacterium typically found in single pairs. It is a prevalent opportunistic pathogen in clinical practice that can cause healthcare-associated infections in both humans and animals, including goat, dog, cat, cow, forest musk deer, mink, blue fox, and so on. *P. aeruginosa* plays a significant role in diseases such as intestinal infections. We confirmed that *P. aeruginosa* was identified as the pathogen in the diarrhea feces of a cynomolgus monkey through morphological analysis, biochemical tests, 16S rRNA gene sequencing, and animal pathogenicity experiments. Additionally, we performed drug sensitivity testing on *P. aeruginosa*. So far, this is the first case of *P. aeruginosa* isolated from the diarrhea feces of a cynomolgus monkey. This study provides an important scientific foundation for isolating and identifying *P. aeruginosa* as well as for preventing and treating bacterial diseases in experimental monkeys.

## 1. Introduction

Macaques, as the animal model closest to human beings, play an important role in basic research and biomedical research [1]. However, macaques living in open environments are susceptible to infections caused by various pathogenic microorganisms, leading to disease development [2]. Intestinal infections can stimulate the gastrointestinal mucosa and impair both secretion and absorption functions of intestinal fluids. The primary clinical manifestation is diarrhea, a prevalent health problem in experimental monkeys [3,4]. Once diarrhea occurs, it will cause malnutrition in macaques, reduce the reproductive rate, and even lead to death, which seriously threatens the feeding and management of primates and is not conducive to the output of high-quality laboratory animals. Bacteria represent one of the main groups of pathogenic microorganisms responsible for intestinal infections in macaques, due to their close evolutionary relationship with humans and due to the similar gut microbiota observed in developing countries, so there is a significant risk of zoonotic transfer from these pathogens [5,6].

*P. aeruginosa* belongs to the genus Pseudomonas and is an aerobic Gram-negative bacillus with strong environmental adaptability. It is widely present in the external environment such as drinking water and soil, as well as in animals and plants [7], and is an important zoonotic pathogen. It can cause infections in humans and animals, and is a major healthcare-associated pathogen worldwide [8,9]. It can result in skin and soft tissue infection [10,11], keratitis [12], pneumonia [13,14], pulmonary cystic fibrosis [15], urinary tract infection [16], bronchiectasis [17,18], bacteremia [19], and other diseases. In the aquaculture industry, contaminated water, cages, and feed tanks serve as reservoirs of *P. aeruginosa* and are the origin of *P. aeruginosa* outbreaks [20,21]. The epidemic outbreak of *P. aeruginosa* not only leads to significant losses in the livestock and poultry industry but also has a considerable impact on the health of experimental and wild animals and their reproductive derivatives. *P. aeruginosa* can infect humans [22], goats (*Capra aegagrus hircus*) [23], dogs (*Canis lupus familiaris*) and cats (*Felis silvestris catus*) [24,25], cows (*Bos taurus*) [26], forest musk deer (*Moschus berezovskii*) [27], mink (*Mustelidae*) [28], and blue fox (*Alopex lagopus*) [29]. Non-human primates (NHPs) such as rhesus monkeys, squirrel monkeys, and African green monkeys are also susceptible to *P. aeruginosa* infection, which may cause bronchitis and myocarditis [30,31,32]. However, to our knowledge, systematic research on the morphology and molecular identification of *P. aeruginosa* isolated from the feces of diarrheal cynomolgus monkeys has not been reported.

## 2. Materials and Methods

To identify potential causes of severe diarrhea in a cynomolgus monkey (*Macaca fascicularis*), the bacteria was identified by morphological examination, physiological and biochemical tests, molecular biological analysis, a drug sensitivity test, and animal pathogenicity experiments.

### 2.1. Samples

The cynomolgus monkey employed in this study originated from Kunming University of Science and Technology [SYXK (Yunnan) K2022-0001]. The clinical manifestations of the monkey were as follows: watery stools, listlessness, dehydration, and decreased appetite. After sterilizing the skin around the anus with iodophor, feces were collected in a sterile 2 mL centrifuge tube via a sterile disposable sampling swab.

### 2.2. Bacterial Isolation and Morphological Observation

Anal swab samples were thoroughly mixed in sterile saline solution. The resultant suspension was inoculated onto LB nutrient agar (Hopebio, Qingdao, China). The plates were incubated at 37 °C for a period of 24 h. Single colonies were selected for streaking and subculturing on acetamide agar medium (Hopebio, Qingdao, China). Additionally, three colonies were isolated and inoculated into 5 mL of LB broth medium (Hopebio, Qingdao, China). The cultures on the plates were maintained at 37 °C for a duration of 18–24 h to observe bacterial growth characteristics. Purified and cultured isolates were collected for smear staining (Gram stain kit, Solarbio, Beijing, China) and observed under a microscope.

### 2.3. Physiological and Biochemical Identification

The colonies to be detected were selected in normal saline, and the bacterial suspension of 0.5 McFarland (approximately 10^8^ CFU/mL) was prepared. Under sterile conditions, 100 µL of bacterial suspension was picked out with an inoculation rod and inoculated into the biochemically encoded identification tube CYZ-15E for the Enterobacteriaceae kit (Binhe Microorganism Reagent, Hangzhou, China) and sealed. Based on the instructions of the kit, the inoculated identification tubes were incubated at 37 °C for 18–24 h and the results were observed. The physiological and biochemical characteristics of the obtained strains were compared with the results of the standard strains in the Manual of Identification of Common Bacterial Systems to preliminarily determine the strain species.

### 2.4. Bacterial 16S rRNA Amplification and Sequencing

Bacterial 16S rRNA gene universal primers were used [33] (27F, 5′-AGAGTTTGATCCTGGCTCAG-3′, and 1492R, 5′-CGGCTACCTTGTTACGACTT-3′) for PCR identification. The PCR reaction system was as follows: 12.5 µL of 2× Taq PCR Master Mix (Tiangen, Beijing, China), the upper and lower primers (10 µmol/L) comprised 1 µL each and were supplemented with distilled water up to a total volume of 25 µL, and a small quantity of bacteria was used as the PCR template. The PCR amplification protocol included an initial denaturation step at 95 °C for 3 min, followed by 35 cycles of denaturation at 95 °C for 30 s, annealing at 55 °C for 30 s, and extension at 72 °C for 1 min. A final extension step occurred at 72 °C for 5 min. The PCR products were identified by agarose gel electrophoresis and recovered, and sequencing was carried out by Beijing Tsingke Biotechnology Co., Ltd., in Beijing, China.

### 2.5. Sequence Alignment Analysis and Construction of Phylogenetic Tree

For sequence alignment analysis and phylogenetic tree construction purposes, the acquired gene sequences underwent homology comparison via BLAST search against related sequences available in NCBI GenBank (MegAlign 7.0.26). Subsequently, MEGA11 software facilitated the construction of a phylogenetic tree aimed at analyzing and comparing the genetic relationships among *P. aeruginosa* strains derived from various sources.

### 2.6. Antibiotic Susceptibility Testing

A drug susceptibility test was performed according to the Kirby–Bauer disk diffusion method. A total of 100 µL of the purified *P. aeruginosa* bacteria suspension was spread on the ordinary agar medium, and the drug-sensitive tablets of 24 antibiotics were pasted on the medium. After 18 h of incubation at 37 °C, the inhibition zone diameter of each antibiotic was measured.

### 2.7. Bacterial Artificial Regression Infection Test

The isolated and purified strain of *P*. *aeruginosa* (PA/CM-101101) was inoculated onto a slant of nutrient agar and incubated at 28 °C for a duration of 18–24 h. Subsequently, the bacterial colony was washed with sterile saline. A total of ten healthy mice aged 9 to 10 weeks were randomly and evenly allocated into an experimental group and a blank control group. Five mice in the experimental group received an intraperitoneal injection of 0.1 mL of bacteria suspension that had been diluted using a 10-fold dilution method from an initial concentration ranging from 1 × 10^9^ to 1 × 10^5^ CFU/mL in sterile phosphate-buffered saline (PBS), while the control group consisted of five mice that were administered the same volume of sterile PBS buffer via intraperitoneal injection. After injecting the bacteria, the disease symptoms and the number of dead mice were observed and recorded every morning and evening. The mice underwent normal feeding management for 7 days.

## 3. Results

### 3.1. Morphological Characteristics of Bacteria

**Figure 1:**
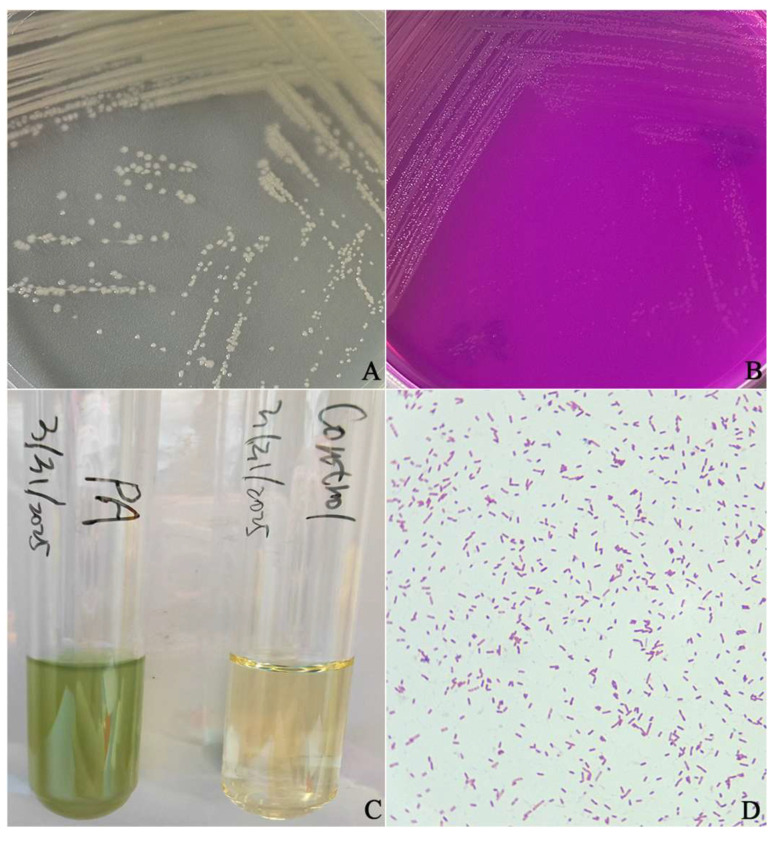
The morphology of *P. aeruginosa* isolated from a cynomolgus monkey. (**A**) Morphological observations of the *P. aeruginosa* isolate on an LB agar plate. (**B**) Morphological characteristics of the isolate on acetamide agar. (**C**) Morphological observations of the *P. aeruginosa* isolate in LB broth. (**D**) Morphological observations of the *P. aeruginosa* and Gram staining microscopy results.

The anal swab was cultured on LB agar medium, revealing colony growth in only one morphological form. The isolated bacteria exhibited smooth, slightly elevated colonies after being cultured on LB agar medium for 24 h, and these colonies had neat and wavy edges (Figure 1A). When transferred to acetamide agar at 37 °C for an additional 24 h, the colonies appeared flat with irregular edges surrounded by slightly pink coloration; concurrently, the color change in the medium resulted in rose-red pigmentation (Figure 1B). Culturing in LB broth for another day led to turbidity accompanied by yellowish-green coloration (Figure 1C). Gram staining results indicated negative reactions characterized by blunt ends without spore formation (Figure 1D).

### 3.2. Results of Bacterial Biochemical Identification

Physiological and biochemical identification revealed that the isolated strain could catabolize glucose and urea, but could not metabolize mannitol or raffinose. The methyl red test, Voges–Proskauer test, and indole test yielded negative results; no hydrogen sulfide production was observed. However, both the motility and the citrate tests revealed positive results, aligning with the known biochemical characteristics of *P. aeruginosa* (Table 1).

### 3.3. 16S rRNA Gene Sequencing, Homology Comparison, and Phylogenetic Analysis

**Figure 2:**
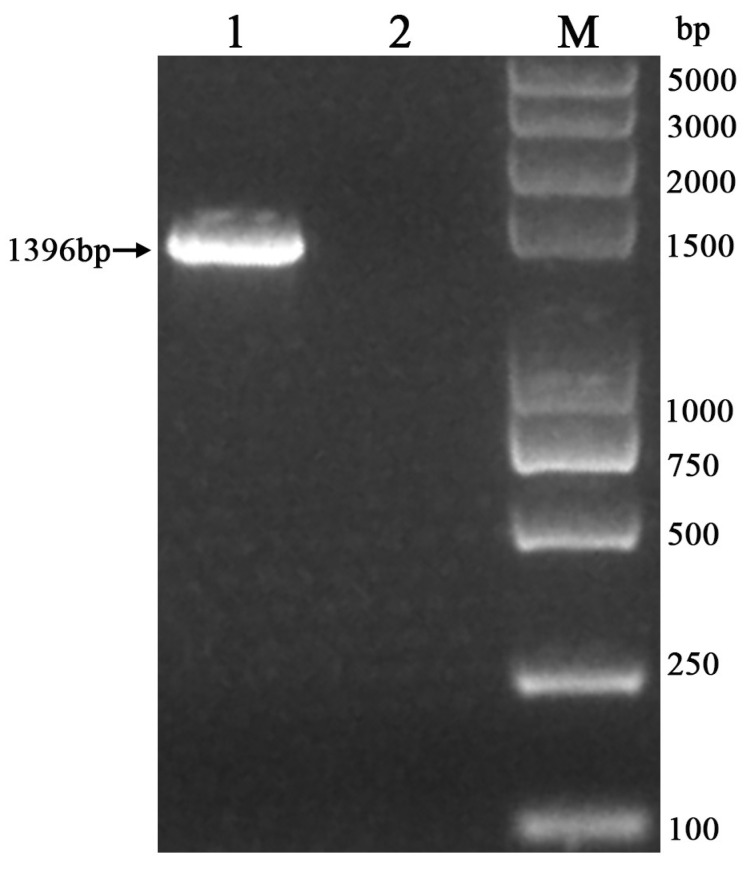
Identification of PCR-amplified *P. aeruginosa* with 16S rRNA gene in a cynomolgus monkey (M: DL-2K marker; 1: sample; 2: negative control).

The amplified PCR products were subjected to analysis via 1.5% agarose gel electrophoresis to isolate the target gene band of the anticipated size (1000–2000 bp) (Figure 2). The sequence of the 16S rRNA gene obtained from the *P. aeruginosa* strain isolated from the cynomolgus monkey was determined to be 1396 bp in length (Supplementary Materials).

**Figure 3:**
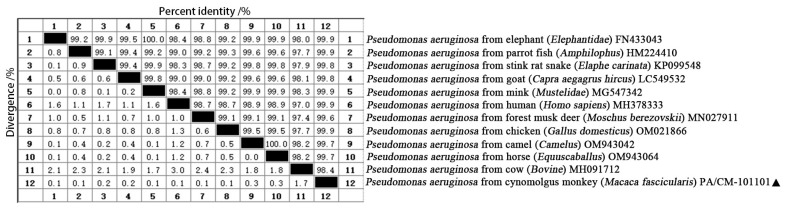
Comparison of similarity between *P. aeruginosa* of a cynomolgus monkey (*Macaca fascicularis*) (PA/CM-101101) and other strains. “▲” is the isolated strain in this study.

The sequencing of the obtained 16S rRNA was conducted using the Sanger dideoxy method, and the results have been reported in the NCBI (GenBank Accession No.: PV608028). Sequence alignment analysis was performed using the Clustalw method, and the results indicated a coincidence rate of over 95% with *P. aeruginosa*, thereby confirming that the isolate was indeed *P. aeruginosa*. The 16S rRNA gene sequence analysis showed that the sequence identity of strain PA/CM-101101 shared more than 98.4% similarity with 11 strains of *P. aeruginosa* from various sources in GenBank, showing maximum homology with elephant (*Elephantidae*) (GenBank Accession No.: FN433043), parrot fish (*Amphilophus*) (GenBank Accession No.: HM224410), mink (*Mustelidae*) (GenBank Accession No.: MG547342), human (*Homo sapiens*) (GenBank Accession No.: MH378333), and chicken (*Gallus domesticus*) (GenBank Accession No.: OM021866) at a similarity rate of 99.9% (Figure 3).

**Figure 4:**
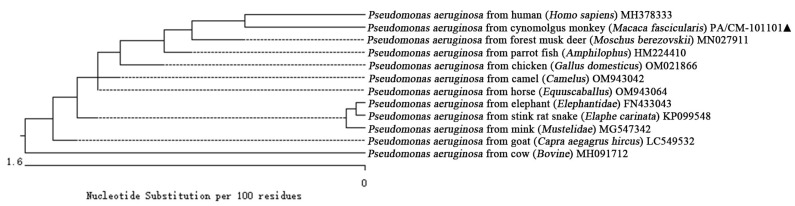
Phylogenetic tree of 16S rRNA sequences among different reported strains of *P. aeruginosa*, including cynomolgus monkey (*Macaca fascicularis*) (PA/CM-101101). “▲” is the isolated strain in this study.

The Clustalw method in MegAlign (version 11.1.0.59) software was used to align the sequences and construct the phylogenetic evolutionary tree. The results indicate that bacterial strain PA/CM-101101 isolated from the anal secretion of the cynomolgus monkey formed a distinct branch alongside human (*Homo sapiens*) (MH378333) within the phylogeny of *P. aeruginosa* (Figure 4). Furthermore, it clustered together with strains from forest musk deer (*Moschus berezovskii*) (MN027911), parrot fish (*Amphilophus*) (HM224410), chicken (*Gallus domesticus*) (OM021866), camel (*Camelus*) (OM943042), horse (*Equuscaballus*) (OM943064), elephant (*Elephantidae*) (FN433043), stink rat snake (*Elaphe carinata*) (KP099548), mink (*Mustelidae*) (MG547342), and goat (*Capra aegagrus hircus*) (LC549532) strains within *P. aeruginosa* phylogeny, while forming a separate branch from that of cow (*Bovine*) (MH091712). Therefore, we conclude that the pathogenic bacterium isolated from the cynomolgus monkey (*Macaca fascicularis*) was indeed identified as *P. aeruginosa* based on 16S rRNA gene sequence analyses. It was most closely related to the *P. aeruginosa* from human (*Homo sapiens*) (MH378333), while it had a distant relationship with *P. aeruginosa* of the cow (*Bovine*) (MH091712).

### 3.4. Antimicrobial Susceptibility Test Results of the Isolated Strain

To evaluate antibiotic sensitivity profiles for strain PA/CM-101101 against 24 different chemical agents, we employed the Kirby–Bauer disc diffusion methodology. The results indicate that this isolated strain exhibited sensitivity towards amikacin, azithromycin, cefoperazone, ceftazidime, ceftriaxone, ciprofloxacin, gentamicin, imipenem, levofloxacin, meropenem, norfloxacin, ofloxacin, and polymyxin B; moderate sensitivity was observed towards amoxicillin, bacitracin, cotrimoxazole, furazolidone, lincomycin, rifampicin; and resistant sensitivity was observed towards ampicillin, cefadroxil, cefazolin, erythromycin, and vancomycin (Table 2).

### 3.5. Results of Animal Pathogenicity Tests

Four hours after the injection of the bacterial liquid, the mice in the experimental group began to show symptoms such as listlessness, slow activity, loss of appetite, and diarrhea, and all died within 48 h. Culture characteristics and morphological features consistent with strain PA/CM-101101 were isolated from the dead mice. All the mice in the control group were healthy for 7 days. No gross lesions were found in the internal organs during post-mortem examination.

## 4. Discussion

*P. aeruginosa* is an opportunistic pathogen in clinical practice that can cause healthcare-associated infections in both humans and animals [34], and can even lead to mortality [35]. It seriously threatens the health of humans and animals. However, recent reports on *P. aeruginosa* have primarily focused on nosocomial infections as well as infections in livestock and poultry. So far, *P. aeruginosa* has not been reported to cause intestinal infection in cynomolgus monkey.

Bacterial diseases spread rapidly and have a high mortality rate, so it is necessary to isolate and identify the pathogenic bacteria in order to study their effects on animal health [36]. Utilizing 16S rRNA molecular markers for bacterial taxonomic identification proves highly effective [37]. In this study, bacterial strain PA/CM-101101 was isolated from the feces of a diarrheal cynomolgus monkey. The morphological characteristics and biochemical identification were consistent with those typical of *P. aeruginosa*. The isolated strain showed high homology with *P. aeruginosa* from different species, and was the closest to the *P. aeruginosa* strain from *Homo sapiens* (MH378333). Further studies are needed to determine whether the genetic characteristics of *P. aeruginosa* are related to different species and sources. Pathogenicity tests conducted on mice showed symptoms such as listlessness, slow activity, loss of appetite, and diarrhea, culminating in mortality within 48 h post-infection, which confirmed that *P. aeruginosa* was the pathogen causing diarrhea in the cynomolgus monkey.

Currently, antibiotic therapy remains the primary treatment option due to its widespread application and rapid efficacy; however, many bacteria have developed resistance against it [38]. Long-term overuse of antibiotics not only results in residues but also fosters antibiotic-resistant bacteria (ARB) and antibiotic resistance genes (ARGs) [39]. It has been reported that *P. aeruginosa* is highly resistant to antibiotics [20,40,41], particularly concerning carbapenem resistance, which poses life-threatening risks that severely impact public health safety [42]. Consequently, in order to develop a more rational use of drugs for *P. aeruginosa*, there is an ongoing effort towards standardizing rational use protocols for antibiotics aimed at minimizing indiscriminate usage while reducing environmental pollution and animal casualties. In this study, we conducted a drug susceptibility test on *P. aeruginosa*, and the results indicate that the isolates exhibited good sensitivity to most antibiotics. We hypothesize that this finding may be related to the frequency of daily antibiotic use at this location. It has certain guiding significance for the breeding and management of cynomolgus monkeys in the future. In the aquaculture industry, contaminated water, cages, feed tanks, etc. are reservoirs of *P. aeruginosa* and are a source of outbreaks of *P. aeruginosa* [20,21]. Therefore, maintaining clean environmental hygiene during the feeding and management of experimental monkeys plays an important role in the prevention of *P. aeruginosa*.

## 5. Conclusions

In conclusion, *P. aeruginosa* was identified as a pathogen in the feces of a diarrheal cynomolgus monkey through morphological analysis, biochemical tests, 16S rRNA gene sequencing, and animal pathogenicity experiments. The isolated strain showed high homology with *P. aeruginosa* from different species, and was the closest to the *P. aeruginosa strain* from *Homo sapiens* (MH378333). The isolated strains demonstrated sensitivity to 13 antibiotics, including imipenem and meropenem. This study provides an important scientific foundation for isolating and identifying *P. aeruginosa* as well as for preventing and treating bacterial diseases in experimental monkeys.

## Figures and Tables

**Table 1:** Physiological and biochemical characteristics of strain PA/CM-101101.

Biochemical Project	Results	Biochemical Project	Results
Mannitol	−	Semi-solid agar (motility)	+
Hydrogen sulfide(H_2_S)	−	Glucose (gas)	−
Phenylalanine	−	Lysine	+
Glucose	+	Ornithine	+
Indole	−	Raffinose	−
Voges–Proskauer test	−	Sorbitol	−
Methyl red test	−	Urea	+
Citrate	+	Xylose	−

Note: +: positive; −: negative.

**Table 2:** Drug sensitivity analysis results of isolated strain.

Antibiotics	Medication Dose (µg/tablet)	IZD (mm)	Sensitivity	Judgment Standard of Inhibition Zone Diameter (mm)		
				Resistant	Medium Sensitivity	Highly Sensitive
Amikacin	30	23	S	≤14	15–16	≥17
Amoxicillin	10	7	I	≤5	6–9	≥10
Ampicillin	10	7	R	≤13	14–16	≥17
Azithromycin	15	28	S	≤12	–	≥13
Bacitracin	0.04	7	I	≤6	7–9	≥10
Cefadroxil	30	7	R	≤13	14–16	≥17
Cefazolin	30	7	R	≤14	15–17	≥18
Cefoperazone	75	24	S	≤15	16–20	≥21
Ceftazidime	30	25	S	≤17	18–20	≥21
Ceftriaxone	30	28	S	≤13	14–22	≥23
Ciprofloxacin	5	35	S	≤15	16–20	≥21
Cotrimoxazole	23.75	11	I	≤10	11–15	≥16
Erythromycin	15	10	R	≤13	14–22	≥23
Furazolidone	300	7	I	≤5	6–9	≥10
Gentamicin	10	15	S	≤12	13–14	≥15
Imipenem	10	28	S	≤19	20–22	≥23
Levofloxacin	5	31	S	≤13	14–16	≥17
Lincomycin	2	7	I	≤5	6–11	≥12
Meropenem	10	31	S	≤19	20–22	≥23
Norfloxacin	10	28	S	≤12	13–16	≥17
Ofloxacin	5	22	S	≤12	13–15	≥16
Polymyxin B	300	14	S	≤8	9–11	≥12
Rifampicin	5	7	I	≤6	7–9	≥10
Vancomycin	30	7	R	≤9	10–11	≥12

Note: S: susceptible, I: intermediate, R: resistant.

## Data Availability

All data in this study are available from the corresponding authors upon reasonable request.

## Supplementary Materials

The following supporting information can be downloaded at: https://www.mdpi.com/article/10.3390/vetsci12070636/s1.
