# Supplementary figures and images for: Isolation, Identification, and Drug Sensitivity Test of Pseudomonas aeruginosa from Cynomolgus Monkey (Macaca fascicularis)
Source: Vet Sci. 2025 Jul 3;12(7):636. doi: 10.3390/vetsci12070636 (PMC12299744; doi:10.3390/vetsci12070636)

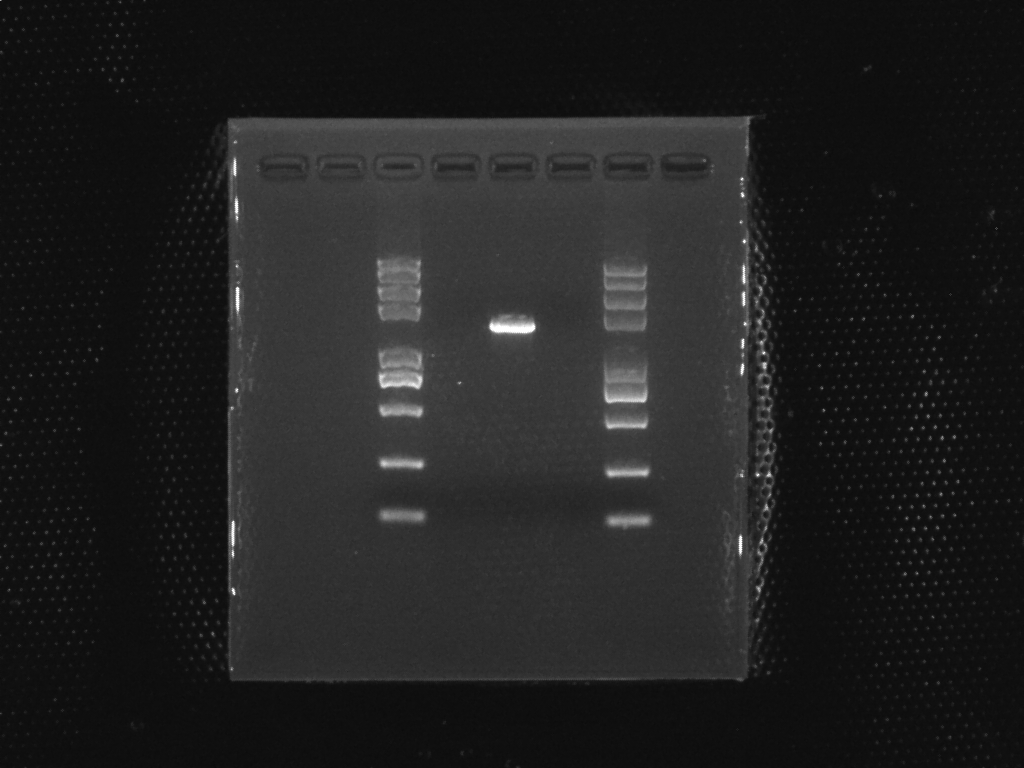

Supplement: Supplementary file 1 [file vetsci-12-00636-s001.zip › vetsci-3695973.jpg]
